# Optimizing Insecticide Application Timing for Broad Bean Weevil Control and Minimizing Crop Damage in Broad Bean (*Vicia faba* Linn.)

**DOI:** 10.3390/plants12091839

**Published:** 2023-04-29

**Authors:** Mohammad Almogdad, Roma Semaškienė, Kęstutis Tamošiūnas

**Affiliations:** Department of Plant Pathology and Protection, Institute of Agriculture, Lithuanian Research Centre for Agriculture and Forestry, Akademija, LT-58344 Kėdainiai distr., Lithuania; roma.semaskiene@lammc.lt (R.S.); kestutis.tamosiunas@lammc.lt (K.T.)

**Keywords:** abundance, bruchid, damaged seed, pest, spray time, *Vicia faba*, yield

## Abstract

During the growing seasons of 2018 to 2020, a field experiment in broad bean (*Vicia faba* L.) was conducted at the Lithuanian Research Centre for Agriculture and Forestry. The objective of the study was to explore the effects of the timing of insecticide application on the abundance, damage, and control of the broad bean weevil (*Bruchus rufimanus* Boh.). The experiment included four spray regimes and an untreated control. Yellow water traps were utilized to monitor the broad bean weevil from germination to senescence. Results indicate that broad bean weevil infestation occurred in all study years, with the highest density of adults observed during the flowering stage. Damage to seeds ranged from 23% to 59.62%. The data suggest that *B. rufimanus* infestation can result in a 19.1% reduction in seed yield. However, spraying when the daily temperature exceeded the threshold for adult activity for 3 days and at the end of flowering produced a significant increase in yield of 13.3% and 6.6%, respectively. Additionally, the spray at the end of flowering reduced damaged seeds by 21.4–48%.

## 1. Introduction

Globally, the broad bean (*Vicia faba* Linnaeus) is one of the main legume plants in agriculture [1]. The broad bean is used as green manure [2] because the root nodules contain symbiotic nitrogen-fixing bacteria that can fix nitrogen by 130 to 160 kg N ha^−1^ [3]. Its importance lies in its high grain protein content, and thus its use as a feed (seed and pods) and food (seed) [4]. The total broad bean yield in 2020 was around 218.6 thousand tons from 58.3 thousand hectares of the sown area in Lithuania, with an average yield of 3.75 t ha^−1^, while it was 3 thousand hectares in 2010 [5]. Climate change is causing changes in pest dispersal and growth rates of the insect population, as well as a longer overwintering period, more generations, and an increase of both insect pests and natural enemy activity. Several studies have found that temperature has an impact on the initiation and termination of diapause [6]. When environmental indicators, like temperature thresholds, are achieved, compulsory diapause may come to an end [7,8]. Changes in species dispersal have occurred as a result of climate change, with many species migrating earlier in recent years than in the past [9]. Lithuania’s average annual temperature has risen by 0.7–0.9 °C in comparison with the 20 year average, which agrees with the work of Ozolinèius [10], which reported that the spring and autumn thermal periods have been longer.

In many regions of the world, including Europe, *Bruchus rufimanus* has recently become the most important insect pest of the broad bean [11,12]. At the onset of the flowering stage, adults start dispersing to broad bean crops from overwintering sites [13] and start laying eggs on pods [14]. After 10 days, the majority of the eggs hatch [15]. Larvae burrow through the pod and into the seed, consuming the endosperm, resulting in lower germination and a yield reduction of up to 70% when compared to the healthy seed [16]. The percentage of the seed destroyed by *B. rufimanus* varies between 18.5 and 28.9%, influenced by meteorological conditions [17]. Furthermore, when compared to the healthy seed, the damaged seed had a 13% reduced germination rate [16]. Larvae spend four instars inside the pods consuming the seed, thereby reducing seed mass and decreasing its utility as feed [17]. With *B. rufimanus* infesting the bean pods, the commercial value of broad bean output is lowered. The maximum percentage of damaged seed allowed in the human consumption market is 3% [18]. For export quality norms, the same infestation rate is permitted [19].

*Bruchus rufimanus* used to be an occasional pest in Lithuania [20], but its incidence has greatly increased in broad bean fields as the sown area has expanded in recent years (since 2014). Different broad bean weevil management techniques have been investigated in relation to sowing date, plant density, and variety used, and have shown significant effects on the percentage of damage by *B. rufimanus* [12]. 

Insecticides are now recognized as the most efficient insect pest control method in legumes [21]. The harmonization of different control measures would contribute to the objectives to reduce the use of insecticides. The emergence and abundance of insect pests should be monitored throughout the growing season in order to ensure that less insecticides are used.

As *B. rufimanus* larvae develop inside the seed, they are difficult to control using insecticides, hence the reason for targeting the adult. To avoid oviposition, insecticide spray should be carried out throughout the mid-flowering and early pod-set development stages [12]. In Europe, the main means of controlling this insect pest are sprays of pyrethroid and neonicotinoid insecticides [18]. Due to the mobility of weevils and the pyrethroid spray breaking down at high temperatures, insecticides have only 50% efficacy against pea weevil (*B. pisorum* L.) [22].

With the increasing cultivation area of the broad bean, the risk of the previously minor pest species (*B. rufimanus*) becoming a major pest is likely to increase. There are few studies focusing on the impact of broad bean insect pest monitoring and treatment timing to achieve successful control. *Bruchus rufimanus* was investigated in this study, and the incidence, impact, and effective control timing were determined.

## 2. Results

### 2.1. Seasonal Abundance of Bruchus rufimanus Adults

**Figure 1:**
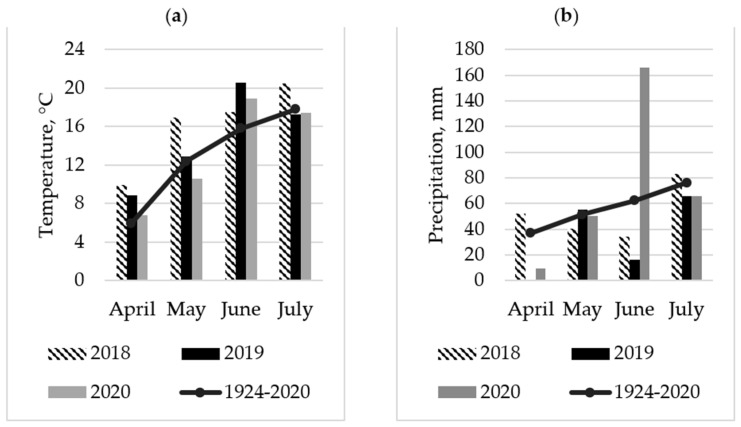
The mean air temperature (**a**) and the total monthly precipitation (**b**) during the 2018–2020 growing seasons and the long-term average (1924–2020).

The growing season of 2018 was much warmer compared to those of the other experimental years, with less precipitation and lower air humidity; hence insects during 2018 were exposed to more favourable conditions compared to other growing seasons (Figure 1). In 2019, the spring period was warm. The beginning of the spring was dry with no rain in April. May was +0.5 °C higher than the long-term average and with an almost similar amount of rain. The average air temperature in June was +5.2 °C warmer compared with the long-term mean. The amount of rain was 74.1% less than the long-term average. In 2020, the summer period was wet. In June, the amount of rain was higher by 166.3% compared to the long-term mean. July was 1.0 °C lower than the long-term mean. The mean air temperature was +3.1 °C higher compared with the long-term mean.

**Figure 2:**
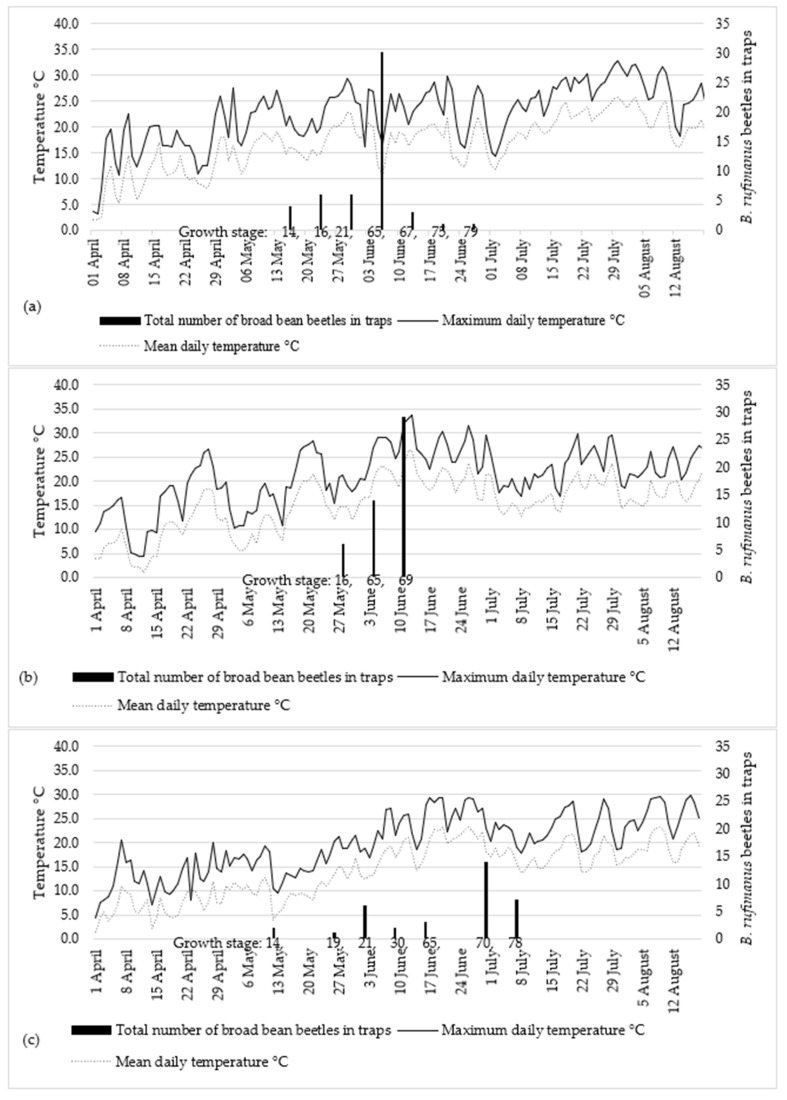
Total *Bruchus rufimanus* adults captured using six yellow water traps placed in the broad bean, during 2018 (**a**), 2019 (**b**), and 2020 (**c**), showing maximum and mean daily temperatures.

Figure 2 shows the total of *B. rufimanus* adults captured in the traps in the 2018, 2019, and 2020 seasons, and the related daily temperature. In 2018, adults of *B. rufimanus* were active from the middle of May until late June. First, *B. rufimanus* beetles (4 adults) were found on 16 May after the accumulation of 336 days of degrees above 5 °C. The mean daily temperature during the week before finding the first adult ranged from 14.7 °C to 19.2 °C. The flowering period of the broad bean extended from 2 June to 15 June. The peak of *B. rufimanus* beetles was observed in the traps during this period (Figure 2a).

In 2019, the first *B. rufimanus* beetles moved to the broad bean field at leaf development stages BBCH 14–16 at 354.6 of days of a base 5 °C temperature. The inflorescence started on 30 May. Later, on 12 June, when the first young pods appeared, *B. rufimanus* abundance peaked when the mean daily temperature had reached 26.4 °C (Figure 2b). After all pods had achieved their maximum length, the number of *B. rufimanus* adults caught in the traps decreased rapidly to zero. 

In 2020, the first *B. rufimanus* beetle was caught on 12 May at growth stage BBCH 14, when the mean daily temperature was 4 °C. The temperature average of the week before 12 May was 9.8 °C and of the week after it was 7.4 °C (Figure 2c). On 30 June, when the first young pods developed and the mean daily temperature was 18.1 °C, the peak of the *B. rufimanus* beetle was observed. The average daily temperature for the week preceding the peak of *B. rufimanus* beetles was 21.5 °C. After all pods had reached the maximum length stage, no further *B. rufimanus* beetles were found in traps.

To check the data pattern for *B. rufimanus* abundance and to examine the effect of air temperature on the movement of *B. rufimanus* from overwintering sites to broad bean fields, a Spearman correlation was carried out (Table 1). Mean daily temperatures were recorded for periods of 2, 7, 14, and 28 days up to the time when the first *B. rufimanus* beetle was caught in the traps. The correlation between the time when the first *B. rufimanus* beetle was caught in the traps and the mean daily temperature of 2-, 7-, and 14-day periods was not statistically significant (*p* > 0.05). For a long period of up to 28 days, it was strong, and, statistically, the correlations were significant (*p* ≤ 0.05). Throughout 2018–2020, during the 28-day period, there were no adults observed at a daily average temperature below 9.7 °C as a daily average temperature. As a result, the emergence of *B. rufimanus* temperature threshold was 9.7 °C.

### 2.2. Bruchus rufimanus Oviposition

**Figure 3:**
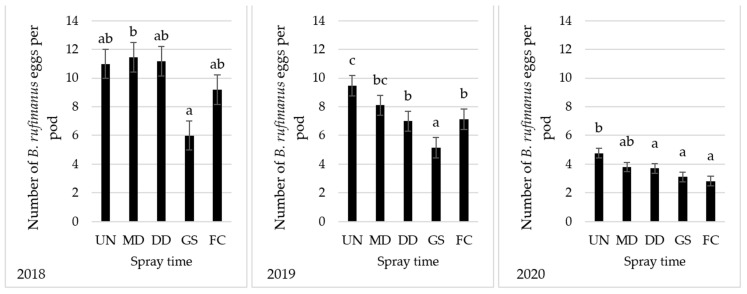
The effect of different spray regimes on eggs laid by *Bruchus rufimanus* on the pods of the broad bean in 2018, 2019, and 2020. Note: Treatments with the same letter are not statistically different according to Duncan’s multiple range test at alpha ≤ 0.05. Key: UN—untreated control. Spray time: MD—when the first *B. rufimanus* weevil was caught in the traps; DD—when the daily temperature for 3 days exceeded that of the threshold (12.5 °C) for adult activity; GS—at the end of flowering; and FC—sprays at three growth stages BBCH 10, 30, and 69.

In 2018, there were significant differences between the plots treated according to the growth stage (GS) at flowering declining and the plots treated when the first weevil was found in the traps (MD), which had almost twice as many eggs (11.4 eggs pod^−1^) as shown in Figure 3. GS treatment gave a lower number of eggs (5.99 eggs pod^−1^) with no significant differences compared to the other treatments (DD and FC) and the untreated control. Full control (FC) spray three times at BBCH 11, 51, and 71 growth stages did not decrease the number of eggs. In 2019, the treatment GS was sufficient to significantly reduce the number of eggs compared to the other spray regimes and the untreated control. The FC treatment, which involved three sprays at BBCH 10, 30, and 69 growth stages, did not provide more benefits compared to the other treatments (MD, DD, and GS) that involved only one spray. This is similar to the results in 2020. The GS treatment significantly reduced the number of eggs by about 34.66% and 18.15% compared to the untreated control and spray regime, respectively, according to monitoring.

### 2.3. Bruchus rufimanus Control

**Figure 4:**
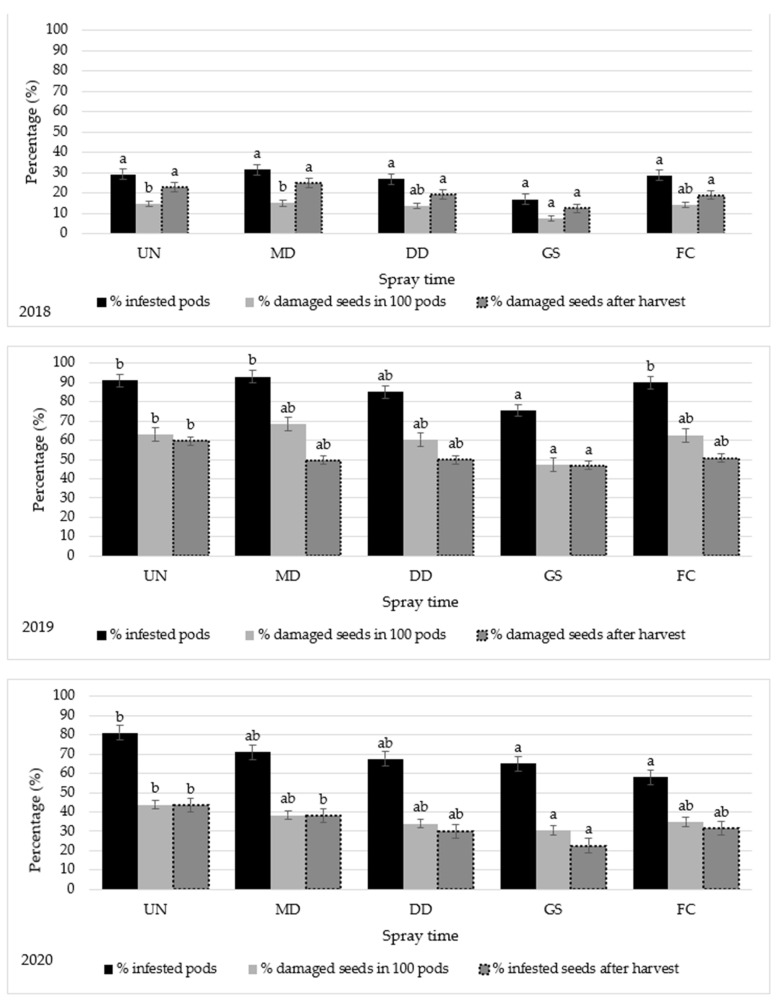
Percentage of *Bruchus rufimanus*-infested pods and seed, and the impact of spray time on the pest damage in 2018, 2019, and 2020. Note: Treatments with the same letter are not statistically different according to Duncan’s multiple range test at alpha ≤ 0.05. Key: UN—untreated control. Spray time: MD—when the first *B. rufimanus* weevil was caught in the traps; DD—when the daily temperature for 3 days exceeded that of the threshold (12.5 °C) for adult activity; GS—at the end of flowering; and FC—sprays at three growth stages BBCH 10, 30, and 69.

In 2018, the percentage of infested pods (PIP) with *B. rufimanus* larvae ranged from 17% to 31.5% (Figure 4). There were no significant differences in PIP values between the treatments. The percentage of damaged seeds (PDS) taken from the tested pods before harvest was reduced significantly in the plots sprayed at the end of flowering (GS) compared to the plots treated when the first weevil was found in the traps (MD) and the untreated control. The lowest PIP and PDS (17% and 7.75%, respectively) were obtained from GS treatment. The additional analyses of seeds after harvest showed that the percentage of damaged seeds after harvest (PDSah) was significantly reduced by the spray regime GS. It was clear that all other spray regimes did not decrease the PDSah compared to the untreated control. In 2019, the treatment GS produced the smallest PIP (75.5%). In comparison to the untreated control, the other spray regimes did not produce significant differences. In 2020, the variations among the treatments were statistically significant. The damage caused by *B. rufimanus* appeared to differ significantly between the untreated plots and treatments at the end of flowering. There were slight insignificant differences in PIP and PDS between the treatments when the first *B. rufimanus* weevil was caught in the traps (MD) (71% and 38.3%, respectively) and the untreated control (81% and 43.7%, respectively). Treatment sprayed at the end of flowering reduced *B. rufimanus* damage significantly compared to the untreated control. The GS treatment produced the lowest PDSah (22.6%) compared to untreated control (43.5%).

### 2.4. Yield

**Figure 5:**
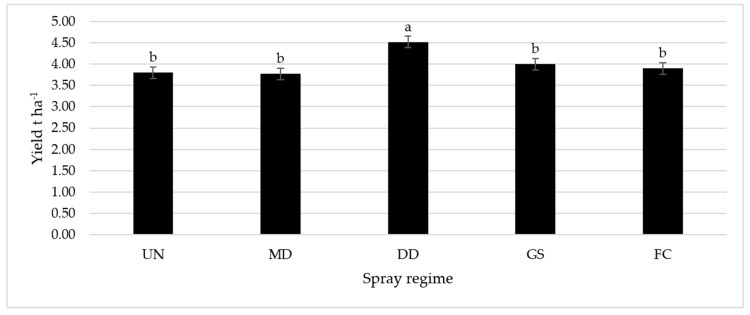
Seed yield average (t ha^−1^) (2018–2020) of broad bean as influenced by the spray regimes against *Bruchus rufimanus*. Note: Treatments with the same letter are not statistically different according to Duncan’s multiple range test at alpha ≤ 0.05. Key: UN—untreated control. Spray time: MD—when the first *B. rufimanus* weevil was caught in the traps; DD—when the daily temperature for 3 days exceeded that of the threshold (12.5 °C) for adult activity; GS—at the end of flowering; and FC—sprays at three growth stages BBCH 10, 30, and 69.

Each year, yield data was analyzed separately, and no spray regime had a significant impact on yield. When all the years of data were combined, the spray regime was found to have a significant factor in yield (Figure 5). The spray when the daily temperature for 3 days exceeded that of the threshold for adult activity (DD) resulted in a considerable yield increase of 19.2% compared to the untreated control. There were no significant differences in yield between other spray regimes (MD, GS and FC) and compared to the untreated control.

## 3. Discussion

Although there have been indications that *B. rufimanus* adults need a certain temperature to colonize crops [18], this has yet to be confirmed in our study. In 2019, the first beetles were captured on May 16 at mean daily temperatures of 14 °C, respectively, which coincided with growth stage BBCH 14. In 2020, the beetles appeared at side shoot formation stages. According to Rusch et al. [23], local climate conditions affect diapause termination and the emergence time of adults. Arrival of the first *B. rufimanus* adults at the fields depended on the daily temperature and growth stage of the host plant [24]. The results showed that adult emergence occurred prior to flowering, in line with the work of Ward [12], which reported that the presence of broad bean flowers was not necessary to affect the termination of diapause and adult emergence. Roubinet [18] and Segers et al. [25] reported that when the daily temperature reaches 15–20 °C, *B. rufimanus* adults start moving from overwintering sites to broad bean fields and feed on nutrients from available flowers of other plants available in the host field area. Medjdoub-Bensaad et al. [26] reported that females of *B. rufimanus* started arriving at broad bean fields at the onset of flowering, while males appeared 1 month earlier during the stem elongation stage. Generally, the density of *B. rufimanus* adults increased gradually and reached peaks at peak bloom. This study showed that *B. rufimanus* had one generation per year over the study years. In our study, the highest abundance was at the full bloom of the broad bean, and this can be explained by the fact that flowers are considered a trophic substrate for *B. rufimanus* adults, as Hamani-Aoudjit et al. [24] reported. Our results agree with those of Titouhi et al. [27], Titouhi et al. [28], and Gebremedhin et al. [29], who all confirmed that the peak of adult activity coincided with the full flowering stage in the host plants. Previous studies showed that during the start of the adult colonization period, the trophic resource abundance determined the adult density [26]. According to Hamidi et al. [30], there was a complete synchronization between the host plant and *B. rufimanus*. The study also suggested that the substantial release of floral scent from the blooming broad bean field could potentially affect the attraction of *B. rufimanus*. The primary factor driving *B. rufimanus* activity was the attraction of the host plant [31]. The phenology of the host plant, which serves as a food supply for *B. rufimanus*, and climate variables are important for its population dynamics [32]. Other studies on the dynamics of *B. rufimanus* occurrence reported that broad bean weevil activity started when the air temperature was higher than 15 °C with maximum activity at full bloom [26], which is in line with our results.

The mean daily temperature had an influence on the beginning of *B. rufimanus* activity and dispersal from overwintering sites. Emergence of the weevils occurred between mid-May and mid-June during the study years between growth stages BBCH 14 and 21 (leaf development stage). Although the optimal temperature for the activity of *B. rufimanus* adults is 15 °C [33], dispersal from overwintering sites in this study occurred at 9.5 °C (mean daily temperature for 7 days prior the monitoring). This is not consistent with what Hoffmann et al. [34] found, as these insects left their overwintering areas toward broad bean crops when the temperature exceeded 15 °C. Although other studies reported that temperature thresholds are required for adults’ emergence [18], our results did not corroborate that. *Bruchus rufimanus* adults were found in the traps before broad bean flowering, and this is consistent with Medjdoub-Bensaad et al. [26], who found that males of *B. rufimanus* colonized the broad bean field during the vegetation phase. The thermal temperature was sufficient to prompt the emergence of *B. rufimanus* beetles from overwintering sites; in all study years, they occurred in broad bean fields prior to the flowering stage. *Bruchus rufimanus* abundance increased at the flowering stage of the broad bean. This is in line with other studies, which found that the beetles of the Bruchinae subfamily were attracted strictly by the volatiles of the broad bean at flowering stage [35]. The results indicated that the peaks of *B. rufimanus* abundance in all the tested broad bean varieties coincided with the end of the flowering stage. The *B. rufimanus* females feed on the plant flowers to promote female reproduction, and their sexual maturity begins in the fields [36]. The results showed that during the pod development stage, the number of *B. rufimanus* beetles captured in the traps started to decrease to the total absence, which could be due to their dispersal to other sites where the broad bean varieties still had flowers or due to their death after reproduction [26]. Similar results were obtained by Hamani-Aoudjit et al. [24], who found that the peak of *B. rufimanus* beetles occurred at the flowering time, and then started to decrease until complete absence.

The results of the insecticide spray more than once indicated no increase in efficacy against the number of eggs per pod if more sprays were done after flowering decline, which is consistent with the conclusion of Roubinet [18]: “The end of blooming … is therefore a threshold marking the end of insecticide spray, which should stop no later than five days after last blooming” (p. 23). Due to the high mobility of weevils, they move through the fields and again reinvade the plots after applying insecticides. As only minor differences were recorded between the spray regime once at the end of flowering and the spray regime thrice beginning at BBCH 69 growth stage, in order to avoid the negative effects of insecticides against pollinators or beneficial natural enemies (parasitoid and predators), spray insecticide once at BBCH 69 growth stage will give an acceptable control.

Percentages of infested pods or damaged seeds before harvest did not show significant differences between the treatments, while estimation of the percentage of damaged seeds after harvest showed significant differences between the treatments and was more proper for the interpretation of results. The comparatively high levels of damage in 2019 and 2020 suggested the high activity of adults resulting from the high daily temperature average in June of 2019 and 2020. High levels of damage in treated plots may have been due to random factors where the rainfall, relative humidity, and temperature are factors affecting infestation by *B. rufimanus* [37]. High relative air humidity and rainfall have a negative impact on oviposition, e.g., washing the eggs from the pod surface, and high temperatures also reduce egg viability [38]. Females laid eggs as soon as the first broad bean pods had formed [26] because they were attracted by kairomone released by the pods [25]. After hatching, larvae penetrate the pods and develop inside the seed [13]. So, the main target of the control is adults because females deposit eggs on the surface of pods [11]. The insecticide sprays should coincide with the egg-laying period to be efficient [24]. The reason why there were no differences between some treatments can be explained by the unsuitable phenological development stage for *B. rufimanus* females for laying eggs at the time of insecticide spray. In our study, treatments applied when the first *B. rufimanus* weevil was caught in the traps (MD) or when the air temperature for 3 days exceeded that of the threshold for *B. rufimanus* activity (DD) was performed early at the vegetative development stage, before the formation of the first pods. As a result, the treatments (MD and DD) applied before pod formation did not show any efficacy for *B. rufimanus* control compared to the other effective treatments or the untreated control. This can be explained by the fact that *B. rufimanus* adults were able to move easily to invade plants again in a few days following the spray of insecticides. In 2019, plants that received insecticide three times (at growth stages BBCH 12–13, 15–18, and 70–71) had a high percentage of damage compared to the untreated control and the other treatments. This may be explained by the negative effect of insecticide application frequency on natural enemies [39] causing sudden outbreaks of insect pests [40]. Several parasites attack species of *Bruchus* genus, e.g., the egg parasitoid *Uscana senex* (Hymenoptera: Trichogrammatidae) [41], and the parasitoid *Triaspis thoracica* (Hymenoptera: Braconidae) attacked 80% of *Bruchus* larvae [42]. The lowest seed damage was achieved by applying insecticide at the end of flowering (GS), which is in line with the work of Segers et al. [25], which found that the best time to control *B. rufimanus* was at the beginning of the pod formation stage. Saeidi and Mirfakhraie [43] also reported that the most effective time to control *B. lentis* was during the early flowering until 2 weeks after. The best spray regime was to apply insecticide at the end of flowering (GS). The content of the damaged seed after harvest from the GS treatment was up to 54.3% lower than from the untreated plots. This agrees with Teferra and Dubale [44], who reported that the best method for *B. pisorum* control was to apply the insecticides starting from the beginning of pod formation.

Spraying at the end of flowering and or when the daily temperature for 3 days exceeded that of the threshold for adult activity had a tangible effect on broad bean yield. When compared to the untreated control, these sprays increased the yield by 6.6% and 13.3%, respectively. The results were in line with Ward and Smart [11], who reported that insecticides should be sprayed once when pods had reached 2 cm or the daily temperature for two successive days had approached that are needed for the *B. rufimanus* activity. This may be explained by the biological characters of *Bruchus* species, which finish their larval instars and the pupae stage within the seed at the warehouses for grain [26]. A full control spray regime and spraying when the daily temperature for 3 days exceeded that of the threshold for adult activity raised the yield in our study. Similar findings were obtained by Saeidi et al. [43], who reported that, compared to the untreated control, spraying twice at early pod formation and again 15 days later resulted in a larger yield. This is not in line with Teferra and Dubale [44], who found that spraying *B. pisorum* once at different times (during flowering, flat podded, or full podded) did not result in a substantial increase in yield. Our findings have potential benefits for reducing the reliance on synthetic pesticides and promoting sustainable pest management practices. Although insecticides have been reported to be the most effective control agents against insect pests in legumes [21], the harmonization of correct control time would contribute to the objectives of the Green Deal strategy, which provide for reducing the use of pesticides by 50% in 2030 [45]. The emergence and abundance of insect pests should be monitored throughout the growing season in order to ensure that this is reached. The precision timing of *B. rufimanus* control contributes to the field of integrated pest management by emphasizing the negative effects of spraying more than once or at inappropriate growth stages on natural enemies and pollinators, and doing this will maximize the benefits while minimizing the insecticides that harm the ecosystem.

## 4. Materials and Methods

### 4.1. Details of the Field Experiment

Field trials were established to monitor the abundance of *B. rufimanus* in relation to air temperature and to evaluate the effect of insecticide spray regimes on the damage caused to the broad bean by *B. rufimanus*. The studies were carried out on the broad bean from 2018 to 2020 on the experimental field in Akademja (Institute of Agriculture, Lithuanian Research Centre for Agriculture and Forestry, Akademija, Kėdainiai distr., Lithuania). The sowing rate was 0.5 million seed per hectare and the required agronomic measures were applied. Plants satisfied their water requirement from rainfall without additional irrigation. The sowing dates were 23 April 2018, 24 April 2019, and 9 April 2020, under the same soil and climatic conditions (loam soil with 4.03% of organic matter, pH 608, K_2_O 190 mg kg^−1^ and P_2_O_5_ 192 mg kg^−1^). The Phenological Growth Stage Key [46,47] was used to identify broad bean growth stages (BBCH). Average means daily temperatures for the months (April, May, June, and July) of the broad bean growth season for each year were obtained from an official meteorological station located about 0.5 km away from the experimental site.

### 4.2. Bruchus rufimanus Monitoring, Collection and Identification

To determine the relationship between the abundance of *B. rufimanus*, six yellow water traps were distributed (6 m apart from each other) in the untreated large plot (10 × 34 m) of the field to detect the appearance time and the abundance of *B. rufimanus*. A few colorless and odorless liquid soap drops were added to water in traps to reduce surface tension. The traps were checked once a week. The water was poured through a fine mesh net, and then the insects were washed with water before being placed in a jar containing 70% ethanol. Using the published classification key [48], the species of the subfamily Bruchinae were identified. To calculate the degree days before *B. rufimanus* activity, the 5 °C degree days base was used, as described in [49]. To determine whether the air temperature had an impact on the emergence of *B. rufimanus* from overwintering sites, daily temperature averages across all years of study were recorded for periods of 2, 7, 14, and 28 days prior to the day when the first *B. rufimanus* beetle was captured in the traps.

### 4.3. Bruchus rufimanus Control

In each year, there were plots 2 × 10 m, with each plot bordered on both sides with a 1.25 m wide untreated strip of broad bean plants. Experiments on the determination of a suitable spray regime against *B. rufimanus* were by the method of random blocks in four replications in a randomized complete block design (RCBD). The spray was done with a wheelbarrow sprayer (one spraying path with five nozzles; a spraying span of 2 m; a type of nozzle flatfan; and a spray pressure of 2.5 bar). The active ingredient lambda-cyhalothrin, a contact-acting pyrethroid insecticide (diluted in 300 L per at a rate of 0.2 L per hectare) was applied in four treatments as follows: (1) MD—when the first *B. rufimanus* weevil was caught in the traps; (2) DD—when the daily temperature for 3 days exceeded that of the threshold (12.5 °C) for adult activity [25]; (3) GS—at the end of flowering; and (4) FC—sprays at 3 growth stages BBCH 10, 30, and 69. Spray times are described in Table 2.

### 4.4. Number of Eggs Laid by Bruchus rufimanus on Pods

**Figure 6:**
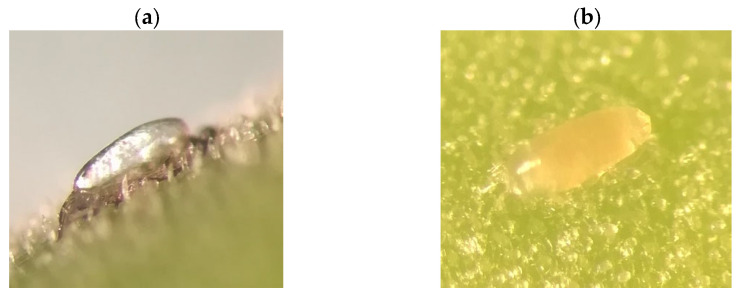
Eggs of *Bruchus rufminaus*—old (**a**), new (**b**).

Oviposition was observed every 5 days between growth stages BBCH 70 and 79. The total number of new and old eggs deposited on the pericarp of pods was recorded during the monitoring phase. During each monitoring, 15 pods located at the base of the plant (formed first) and 15 pods located at the middle of the plant (formed later) from 15 plants were taken randomly from each plot. The sampled pods were checked at the laboratory under binoculars to count the eggs. The old eggs were transparent white, while the new eggs could be distinguished by the yellow colour containing the yolk or larva [25] (Figure 6).

### 4.5. Damaged Seed by Bruchus rufimanus Larvae

**Figure 7:**
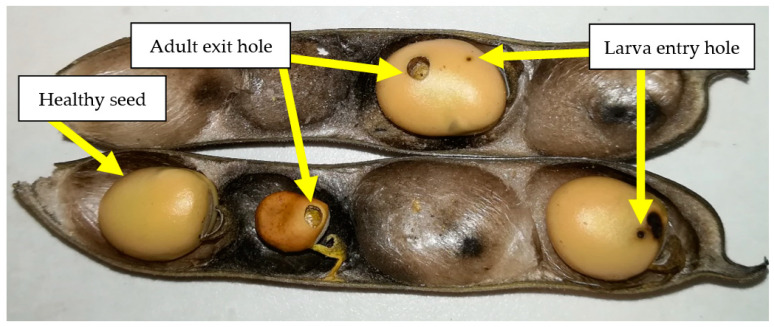
Symptoms of infested broad bean seeds by *Bruchus rufimanus*.

A total of 100 pods from 50 plants (2 pods per plant) were randomly selected from each plot at the completely ripe stage (BBCH 89) before harvest to calculate the percentage of the infested pods. Each pod was opened in the laboratory, and the number of healthy and seeds damaged by *B. rufimanus* were counted (Figure 7). A total of 200 seeds from each plot were randomly selected after harvest to estimate the percentage of the damaged seed.

### 4.6. Yield

The fresh seed weight (kg plot^−1^) was recorded. The yield was adjusted to the moisture content using this formula:

Wd = Ww × [100/(100 − %Moisture)];

where Ww is the weight at the time for which the moisture content was calculated and Wd is the weight after drying. it was then converted to tons per hectare (t ha^−1^).

### 4.7. Statistical Analysis

SAS, version 7.15, was used to record and statistically analyze the data (SAS Institute Inc., Cary, NC, USA). Prior to analysis, the data were examined for homogeneity using the Kolmogorov–Smirnov Test. Analysis of variance (ANOVA) was carried out for the data to determine whether there were differences between the spray regimes. Duncan’s multiple range test was used to determine the significance of differences at alpha ≤ 0.05. The correlation between the air temperature and the abundance of *B. rufimanus* beetles in the traps was analyzed using the Spearman’s correlation analysis.

## 5. Conclusions

Broad bean weevil (*Bruchus rufimanus*) started dispersing to host plant fields at the early leaf development stage (BBCH 14). In all experimental years, the density of adults increased gradually during season and reached peaks at the flowering stage. Over the three seasons, *B. rufimanus* was found to be the most destructive pest to the host plant, with yield losses averaging 19.1% and the content of damaged seed ranging from 23 to 59.62%. The best control of *B. rufimanus* was achieved by the spray when the daily temperature for 3 days exceeded that of the threshold for adult activity or by the spray at the end of flowering resulting in a considerable yield increase (13.3 and 6.6%, respectively). The spray at the end of flowering decreased the damaged seed by 21.4–48%.

## Figures and Tables

**Table 1:** Spearman’s correlation for *Bruchus rufimanus* beetles in the traps with different mean daily temperature periods in 2018–2020.

Mean Daily Temperatures Observed (°C)	Spearman’s Correlation Coefficients	*p* Value
2 days prior to monitoring	0.15378	0.742
7 days prior to monitoring	0.43842	0.3251
14 days prior to monitoring	0.64908	0.1147
28 days prior to monitoring	0.78357	0.0071

**Table 2:** Growth stage (BBCH) and date of application for control *Bruchus rufimanus* on the broad bean from 2018 to 2020.

Spray Regime	Year		
	2018	2019	2020
UN	–	–	–
MD	BBCH 14—16 May	BBCH 15—23 May	BBCH 16—26 May
DD	BBCH 51—28 May	BBCH 65—4 June	BBCH 16—28 May
GS	BBCH 67—14 June	BBCH 69—14 June	BBCH 69—21 June
FC	BBCH 11—7 May	BBCH 13—17 May	BBCH 10—3 May
	BBCH 51—28 May	BBCH 15—23 May	BBCH 16—28 May
	BBCH 71—21 June	BBCH 70—12 June	BBCH 69—11 June

## Data Availability

Not applicable.
